# Effects of age and cognitive function on data quality of standardized surveys in nursing home populations

**DOI:** 10.1186/s12877-019-1258-0

**Published:** 2019-09-03

**Authors:** Patrick Kutschar, Martin Weichbold, Jürgen Osterbrink

**Affiliations:** 1 Paracelsus Medical University, Institute of Nursing Science and Practice, Strubergasse 21, 5020 Salzburg, Austria; 2 Department of Sociology, Paris Lodron University Salzburg, Salzburg, Austria

**Keywords:** Nursing home, Cognitive impairment, Data quality, Measurement error, Item nonresponse

## Abstract

**Background:**

Data quality is of special concern when it comes to survey research in nursing homes. Very little is known about specifics of cognitively impaired elderly in responding to survey questions. This study examines effects of cognitive impairment, age, gender, and interview duration on the data quality in a sample of 659 nursing home residents (NHR).

**Methods:**

Within a cross-sectional design, survey methodology was used to evaluate the pain situation in 13 nursing homes. Residents were stratified into NHR with no/mild (Mini-Mental State Examination MMSE: 18–30) and NHR with moderate (MMSE: 10–17) cognitive impairment. Data quality is measured by item nonresponse (INR). Correlation analyses, ANCOVA, linear and logistic regression models are applied.

**Results:**

Neither interview duration nor gender have effects on item nonresponse. Age accounts for higher INR (β = 0.12, *p* < 0.001). Cognitive impairment strongly predicts INR (β = − 0.40, *p* < 0.001). INR significantly differs between NHR with no/mild (3.98%) and moderate cognitive impairment (11.85%). The likelihood of INR > 5% for residents with moderate cognitive impairment is 3.8-times (*p* < 0.001) of that for those with no/mild impairment.

**Conclusions:**

Surveys are adequate for residents with no/mild cognitive impairment but data quality is threatened in residents with moderate impairments. Precision and validity of responses from NHR with progressed cognitive impairment are potentially limited and results may be biased. The results clearly do support the need for a multidisciplinary ‘general theory’ of the question−/answer-process which has to be also inclusive for cognitively impaired elderly persons.

**Electronic supplementary material:**

The online version of this article (10.1186/s12877-019-1258-0) contains supplementary material, which is available to authorized users.

## Background

Globally, dementia is seen as the most common disease triggering cognitive decline in nursing homes (NH), and around two thirds of German nursing home residents (NHR) are assumed to be affected [1, 2]. During the last decade, there has been a (re-)emerging engagement in understanding the abilities of cognitively impaired elderly. Efforts also comprise strategies to include affected persons in empirical research. Special attention is given to the inclusion of elderly with cognitive impairments (CI) in surveys and its consequences for data quality, i.e. accuracy, consistency and validity [3–6]. However, still little is known about the quality of survey data in NHR. As to conducting survey research among this population, much effort has to be put into the underlying cognitive processes which take place when answering survey questions. It is well documented that the question-answer-process requires several challenging cognitive tasks [7–9]: Respondents have to understand and interpret what is being asked for. They must retrieve information reflecting opinion, behavior, or factual knowledge. An answer has to be generated by retrieving previous judgments and knowledge from memory or computing new judgments ‘on the spot’. This judgment has to be formatted and edited in the sense of the given response alternatives. Before communicating, the answer may be altered to fit the subjective perceived social context of the interview situation. While this question-answer-process has been systematically discussed over the last two decades in general, relatively little is known about the possible obstacles when conducting surveys with cognitively impaired elderly. However, it can be stated that individuals’ ability to respond accurately to survey questions is highly moderated by their cognitive function. Typical consequences of CI or dementia like deterioration in memory, instability of emotional states, decreasing ability to communicate and limited means of comprehension and judgment [10, 11] potentially affect each stage of the question-answer-process. Here, the validity of information obtained by self-report must be questioned: While it is obvious that persons with severe CI are hardly able to self-report even on extremely simplified questions, the chances to obtain reliable and accurate answers from persons with mild to moderate CI have to be discussed.

Obtaining valid, reliable and accurate information from persons with CI is generally a matter of great importance in health services research. When it comes to decisions regarding long-term care, special relevance has to be given the appraisal of responses of those cognitively impaired. Next to the question to which extent affected persons should be involved in care decision making [12–15], strategies to obtain valid information against the background of pain assessment in NHR are being heavily discussed recently [3, 5, 16–18]. One major concern persists in the question, until which level of cognitive decline the so-called gold standard of self-report is still feasible, and at which point proxy assessment should be used. Many results suggest that self-report can be conducted for NHR with up to moderate CI [5, 12, 19, 20]. However, most hypotheses and findings regarding the quality of answers of cognitively impaired elderly still await repeated testing and further evidence.

### Purpose and hypotheses

Methodological research in gerontology, nursing science or social sciences has repeatedly acknowledged a relationship of (age-related) cognitive decline and response quality. Present study is one of the very rare studies to examine the quality of survey responses in NHR with CI against the background of pain and pain management. The purpose was to analyze effects of cognitive impairment, age, gender, and interview duration on data quality in a sample of 659 NHR. Item nonresponse (INR) serves as an indicator for data quality. INR means missing information for a specific variable for a specific respondent. A certain answer may not be provided by respondents (e.g. don’t know, refusal) or is not usable (e.g. answer not possible or inappropriate). INR is a threat to survey quality, a significant problem for survey research, and a substantial source of reduction of precision and generalizability of results [21–23]. Mainly due to restricted cognitive functions and limited means in memory, information retrieval and question comprehension, growing percentages of INR were expected with rising age and the decrease of cognitive function. Topic sensitivity may induce gender-specific question-answer-processes especially when personal and rather intimate questions about pain are asked. It was hypothesized that female respondents refuse to provide answers to potentially sensitive and intimate pain questions more often than male respondents. Interview burden and cognitive demands may increase with the length of the interview. Hence, INR was expected to increase with longer interview duration.

Overall it was hypothesized that proportions of INR increase with rising age, with the decrease of cognitive function, with being female and with interview duration.

## Methods

### Setting and design

Data come from a quasi-experimental study in nursing homes as one part of the large-scale health services research project ‘Action Alliance Pain-free City Muenster’ [24]. A pre-post-test-observational-study was conducted to assess the pain situation and the pain management in 13 out of 32 nursing homes (NH) in the middle-sized city of Muenster (North Rhine-Westphalia*,* Germany). After the intervention phase, same procedures as in the pre-test were applied for the post-test. Cross-sectional data from both samples are used for present secondary analyses.

### Sample and participants

Sampling frame consisted of all NHR with or without any type of pain occurrence who were permanently registered in the facilities, being ≥65 years of age, and provided written informed consent by themselves or by their legal guardians. Exclusion criteria were insufficient German language skills, life-threatening situations, seriously derogated states of health and short-term care. NHR were stratified into three groups of CI using the Mini-Mental State Examination (MMSE) [25]. MMSE estimates cognitive function relating to orientation ability, memory performance, comprehension capability, visual construction and language usage. The measurement results in a score ranging from 0 to 30 points with lower scores indicating more severe impairments. NHR with no or mild impairment (MMSE 18–30) were interviewed with questionnaires, residents with moderate impairments (MMSE 10–17) were examined using both self-report as well as proxy assessment [26]. Residents with severe CI (MMSE < 10) were not examined using survey methodology and therefore were excluded for the current secondary analysis.

### Data collection and measures

Pre-test data collection was conducted from September 2010 until April 2011. After the intervention phase, the second evaluation took place between July 2012 and April 2013. In each NH, data collection was carried out over a period between 4 and 6 weeks, depending on the size and number of eligible NHR of the facility. NHR were interviewed face-to-face using self-report questionnaires (Computer-assisted personal interviewing – CAPI) or observed with proxy assessment tools, depending on the severity of NHR’ state of cognitive impairment. Data collection was executed by 34 specifically trained and qualified research assistants, who had to be either nursing care professionals or students of nursing science with experiences in dealing with elderly persons with CI. All of the research assistants were externs to the nursing homes. The research assistants were obliged to follow a standardized interview protocol and used pre-programmed survey netbooks to collect and record data. All residents obtained printed versions of the questionnaire in order to read along while each question and the respective answer options were read aloud by the research assistants.

### Resident data and questionnaire

Demographical and medical data (e.g. age, sex, diagnoses) were collected from the residents’ medical records. NHR with MMSE≥10 were interviewed with a standardized questionnaire which consisted of 32 questions [see Additional file 3: Questionnaire]. The questionnaire comprised different dimensions of NHR’ pain and health situation (pain localization, pain intensity, duration of pain, state of health) and aspects of perceived pain therapy (received pain medicine, side-effects, non-drug treatments, satisfaction with pain therapy). A multidisciplinary research team developed the questionnaire, whereby validated measures (VRS-5 [27] – verbal rating scale for pain intensity, EQ-5D-3 L [28] – health related quality of life state) were included and further topic-based questions were added. Special focus on survey features (e.g. simplified questions, unidirectional response choices, limited 3-day time-frame on behavior report) was placed. The questionnaire was piloted and adapted before the first evaluation in a comparable NH sample.

### Measurement of results

Primary outcome is the proportion of item nonresponse (INR). The absolute number of administered questions was counted for each NHR. INR was defined either as category ‘I don’t know’ (DK) or ‘cannot be answered’ (CA). DK was an active response given by the NHR, CA a passive response rated by the research assistant, if NHR couldn’t give a substantial answer, couldn’t distinguish between answer categories or stated ‘cannot say’. INR was computed as each data cases’ absolute number of items with DK−/CA-nonresponse in relation to the individual number of administrated questions. Theoretically, INR ranges from 0 to 100%. CI, age, gender, and interview duration are used as explanatory variables. Cognitive impairment is introduced into two ways to measure its influence on INR: a) as metric MMSE score (range 10–30) and b) dichotomized into MMSE-groups (moderate CI/no or mild CI). Age and gender of NHR was collected from medical records. Interview duration was computed automatically based on survey start and end times.

### Statistical analysis

Statistical analyses were conducted using IBM SPSS 24. Sample characteristics were analyzed applying common univariate statistics. Hypothesis testing for differences was conducted using parametric Student t-test for independent samples and analysis of covariance (ANCOVA) was applied for adjustment of mean differences between MMSE-groups. Proportions were tested with Pearson χ^2^-test. For correlation analysis of non-normal metric data, Spearman rho (r_s_, *ρ*) was used. Eta^2^ (ɲ^2^) was computed for measurement of associations between nominal and metric variables. Binary logistic regression (BLR) was applied to test odds ratios (OR) for dichotomous variables MMSE-group and INR (no vs. > 0, > 5, > 10%). Multiple linear regression (MLR) was executed to measure the collective influence of explanatory variables MMSE score, age, gender, and duration of interview on INR, introducing dichotomous pre- versus post-test as control. For mean differences, effect size Cohen’s d (δ; 0.20 < δ < 0.50 small, 0.50 ≤ δ < 0.80 medium, δ ≥ 0.80 large effect) for unequal sample sizes was calculated. Type one error was set to α = 0.05 (two-sided). Bonferroni correction method (adjusted alpha level with k = number of independent statistical tests, α_corr_= $$ \raisebox{1ex}{$a$}\!\left/ \!\raisebox{-1ex}{$k$}\right. $$) was applied to control error inflation. For instance, α_corr_ for the null-hypotheses families regarding the primary outcome INR was set to α_corr_ = 0.00357 (k = 14).

### Ethical clearance

The responsible ethics committee (Medical faculty of Westfälische Wilhems-Universität, Germany) gave ethical clearance (ref. 2010–010-f-S). Participants–or legal guardians of participants–were informed about anonymity and their unconditional right to withdraw from the study. Written informed consent was collected from all participants or their legal guardians.

## Results

### Demographics

**Fig. 1:**
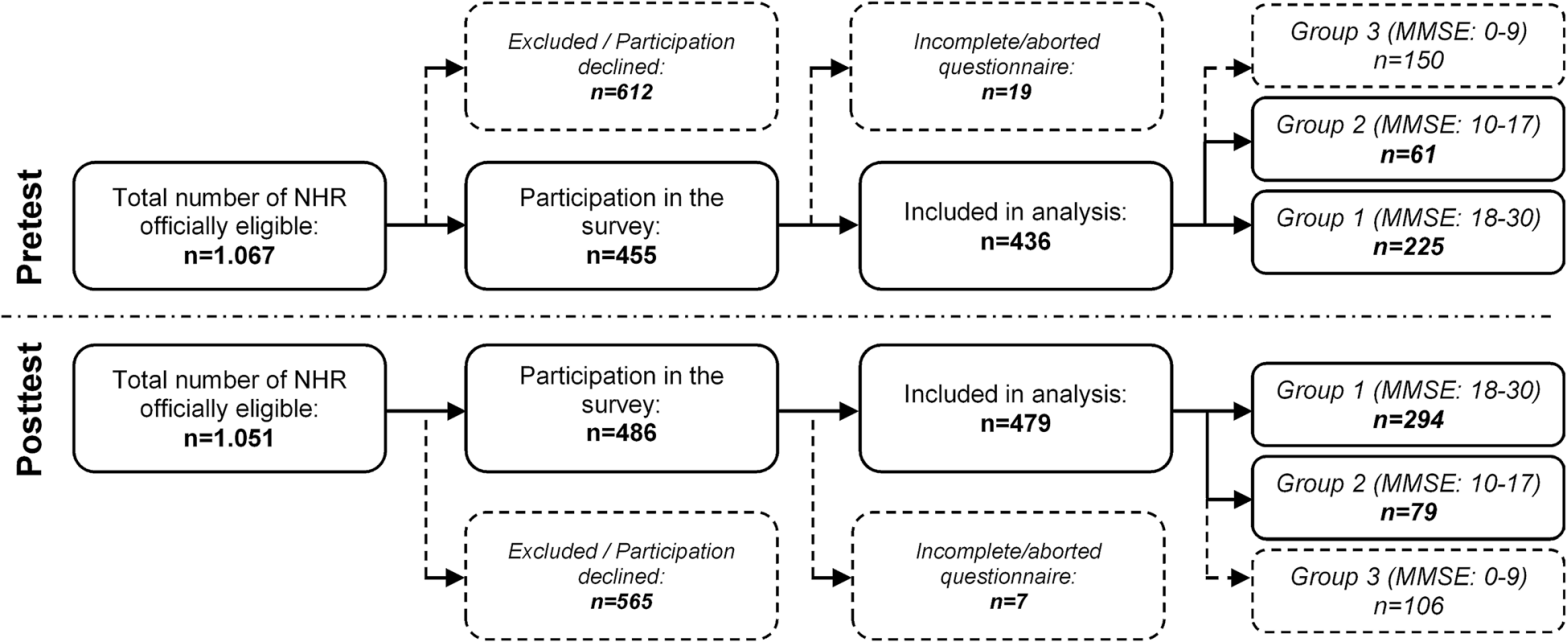
Flow chart of response rates for pre- and post-test

For the pre-test (Fig. 1), a total of 1067 NHR was registered as being consultable for participation. One hundred thirty residents were excluded according to exclusion criteria and 482 residents declined participation. From the 455 NHR participating in the study, 19 data cases were deleted due to incomplete questionnaires (e.g. dropout/abortion, acute deterioration of NHR’ health state, missing medical records). Finally, 436 NHR were included and screened with MMSE, resulting into 150 residents with severe, 61 residents with moderate, and 225 residents with no or mild cognitive impairment. A total number of 1051 NHR was reported to be consultable for post-test (Fig. 1). Exclusion criteria reduced the total number by 396, while 169 residents declined participation. Out of 486 NHR, 7 data cases were identified as incomplete. The included 479 NHR were stratified into 106 residents with severe, 79 residents with moderate, and 294 residents with no or mild cognitive impairment. Response rates were comparable for pre-test (40.9%) and post-test sample (45.6%). Total sample was constructed by merging pre-test and post-test data (i.e. samples are treated as independent measures for INR analyses) and comprises altogether 659 NHR: 519 residents with an MMSE score between 18 and 30, and 140 residents with an MMSE score between 10 and 17.

Table 1 shows the characteristics of participants in detail for total sample, for pre-test and post-test sample as well as for MMSE-group samples. Among the total sample of 659 NHR, three quarter were women. On average, the respondents were 84.5 years of age and have been living in the NH for 3.0 years at the time of the interview. The three most common diagnoses were dementia (31.9%), coronary heart diseases (19.8%) and depression (18.1%). The average MMSE score for the total sample was 21.9. The interviews with respondents lasted 20 min on average. The results of all statistical comparisons between the different samples are displayed in Additional file 1: Table S1. In general, the distributions of sample characteristics are quite similar. Except for varying descriptive distributions of some diagnoses, there are no significant differences in gender, age, duration of stay, and duration of interview—neither between the two groups of cognitive function within each evaluation, nor between the MMSE-groups of pre- and post-test, nor between pre- and post-tests’ total sample distribution.

**Table 1:** Characteristics of participants for pre-, post- and total sample

Characteristics [mean (SD), % (n)]					
		MMSE (18–30)	MMSE (10–17)	Pre−/Post-test	Total
Female	Pre	72.0% (162)	72.1% (44)	72.0% (206)	74.8% (493)
	Post	77.6% (228)	74.7% (59)	76.9% (287)	
Age (years)	Pre	83.35 (7.9)	84.70 (7.3)	83.64 (7.7)	84.50 (7.4)
	Post	85.13 (7.0)	85.27 (7.3)	85.16 (7.1)	
Length of stay (years)	Pre	3.05 (4.0)	4.00 (5.6)	3.25 (4.4)	2.96 (4.2)
	Post	2.84 (4.3)	2.37 (2.8)	2.73 (4.0)	
MMSE score	Pre	23.86 (3.7)	13.98 (2.3)	21.75 (5.3)	21.91 (5.3)
	Post	24.23 (3.5)	13.82 (2.2)	22.03 (5.4)	
Interview duration (min.)	Pre	0:17 (0:22)	0:17 (0:43)	0:17 (0:28)	0:20 (0:41)
	Post	0:25 (0:53)	0:13 (0:24)	0:23 (0:48)	
Diagnoses^a^	Pre	CHD 23.1% (52) DEM 21.8% (49) DJD 17.8% (40)	DEM 42.6% (26) CHD 22.9% (14) APO 16.0% (10)	DEM 26.2% (75) CHD 23.1% (66) DJD 16.8% (48)	DEM 31.9% (210) CHD 19.8% (129) DEP 18.1% (119)
	Post	DEM 29.9% (88) OST 23.5% (69) APO 17.3% (51)	DEM 59.5% (47) DEP 26.6% (21) CHD 21.5% (17)	DEM 36.2% (135) OST 20.4% (76) APO 17.7% (66)	
Sample sizes		n_pre_ = 225 n_post_ = 294	n_pre_ = 61 n_post_ = 79	n_pre_ = 286 n_post_ = 373	*n* = 659

*APO* Apoplex, *CHD* Coronary Heart Disease, *DEM* Dementia, *DEP* Depression, *DJD* Degenerative Joint Disease, *OST* Osteoporosis, *MMSE* Mini-Mental State Examination; ^a^Multiple responses, three most frequent diagnoses are displayed; Bonferroni correction (α_corr_) between pre/post-test within both MMSE-groups α_corr_ = 0.00625 (k = 8), between pre/post-test in entire sample α_corr_ = 0.0125 (k = 4)

### Effects of cognitive impairment, age, gender, and interview duration on INR

The hypotheses dealing with the dependency of INR on the grade of cognitive impairment, age, gender, and length of the interview are tested with different analytic strategies:

(1) The distribution of INR is presented descriptively for pre-test, post-test and total sample as well as its distribution within the two MMSE-groups. (2) Differences in INR between MMSE-groups are analyzed and displayed for pre-test, post-test and total sample. (3) Single correlations of INR with the explanatory variables are presented and the collective effect of the explanatory variables is measured within total sample.

#### Descriptive distributions of item nonresponse rate

**Fig. 2:**
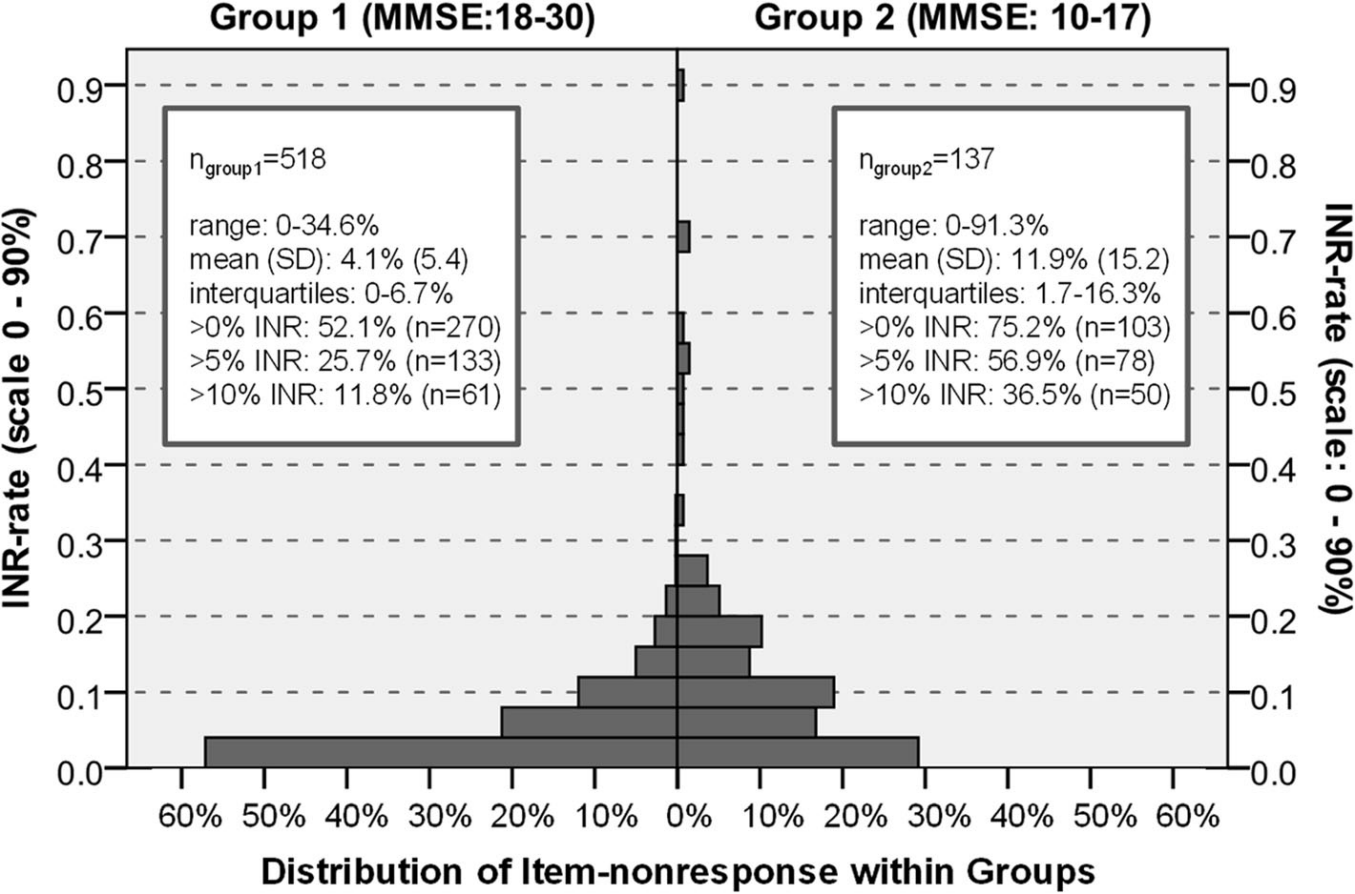
Distribution of item nonresponse within MMSE-groups for total sample

Descriptive statistics of INR are displayed in Additional file 2: Table S2Overall, INR are relatively similar, whereby slightly more NHR with very high INR are observed within the post-test. In total, the INR varies between 0 and 91.3%. NHR weren’t able to state a substantial answer by 5.7% on average (SD 8.9), with a median of 3.9% (IQR 0–8.3%). Pre-tests’ mean INR was 6.1% (SD 9.5), ranging from 0 to 70.4% (IQR 0–8.7%). Post-tests’ INR was 5.4% on average (SD 8.6), with a range from 0 to 91.3% (IQR 0–8.0%). INR univariate statistics for MMSE-group 1 and MMSE-group 2 are displayed in Fig. 2. Within 518 NHR with a MMSE-score between 18 and 30 points, INR ranges from 0 to 34.6%. On average, the proportion of item nonresponse is 4.1% (SD 5.4). Every second resident gave 100% substantial, valid answers. INR of 5% and more was observed in only one quarter of participants. Within the 137 NHR with a MMSE-score between 10 and 17 points, INR was 11.9% (SD 15.2) on average and a range from 0 to 91.3% were observed. Three quarter of NHR were affected of INR in general and almost 60% were affected of at least 5% non-response.

#### INR mean difference between residents with no/mild and moderate cognitive impairment

Comparisons of INR between NHR with no or mild and NHR with moderate CI revealed significant and medium to large effects (Table 2). Residents with moderate CI gave substantially less valid answers than residents with no or mild cognitive impairment. CI_95%_-estimates for INR range 3.3–4.7% (MMSE 18–30) and 10.3–13.0% (MMSE 10–17). The mean difference in INR between the two groups of cognitive function is 7.7 percentage points (*p* < 0.001, *n* = 636) and demonstrates a CI_95%_ population estimation range of difference between 6.1 and 9.2%. Analyses for pre- and post-test samples feature similar results. Overall, ANCOVA show highly significant differences in the mean proportions of INR and strong effects are observed after adjusting for pre−/post-test, sex, age, and duration of interview.

**Table 2:** Comparisons of mean differences in item nonresponse between MMSE-groups

Mean differences for item nonresponse rate between MMSE-groups					
Adjusted comparisons of item nonresponse rates (ANCOVA)					
Sample	Cognitive impairment	Mean (SE, n_valid_)	Mean diff. (SE) [95% CI]	η^2^ (F), p	δ
Pre^a^	No or mild (MMSE 18–30)	4.05% (0.6, *n* = 213) 12.17% (1.1, *n* = 59)	8.13% (1.2) [5.73, 10.53]	14.2% (44.3) *p* < 0.001	0.82
	Moderate (MMSE 10–17)				
Post^a^	No or mild (MMSE 18–30)	3.93% (0.5, *n* = 289) 11.24% (0.9, *n* = 75)	7.31% (1.1) [5.22, 9.40]	11.7% (47.5) *p* < 0.001	0.73
	Moderate (MMSE 10–17)				
Total^b^	No or mild (MMSE 18–30)	3.98% (0.4, *n* = 502) 11.85% (0.7, *n* = 134)	7.67% (0.8) [6.10, 9.24]	12.9% (92.4) *p* < 0.001	0.77
	Moderate (MMSE 10–17)				

*MMSE* Mini-Mental State Examination, *ANCOVA* Analysis of Covariance; n Sample sizes, *SE* Standard error, 95% CI Confidence interval; F ANCOVA statistic; η^2^ partial eta-square; δ Cohen’s d (0.20 < δ < 0.50 small, 0.50 ≤ δ < 0.80 medium, δ ≥ 0.80 large effect); ^a^ adjusted for sex, age, duration of interview; ^b^ adjusted for sample (pre-test/post-test), sex, age, interview duration; α_corr_ ≈ 0.0039 (k = 13)

#### Correlations, BLR and MLR measuring the effects of MMSE, age, gender, and duration

The influence of MMSE, age, gender, and duration of the interview on item nonresponse rates is measured in the total sample by a two-way strategy: by exploring single correlations as well as by measuring the collective explanatory potential in regression models. Correlation analyses (Table 3) show that INR is negatively correlated with MMSE (r_s_ ≈ − 0.36, *p* < 0.001, *n* = 659), and positively correlated with age (r_s_ ≈ 0.16, *p* < 0.001, *n* = 657). INR increases with decreasing levels of CI, as well as with increasing age, respectively. While the effect of MMSE on INR is medium-to-high-ranged, only a weak correlation between MMSE and age can be stated. Neither the correlation of INR and interview duration (r_s_ ≈ 0.07, *p* ≈ 0.089, *n* = 640), nor INR and residents’ gender (ɲ^2^ = 0.0046, *p* ≈ 0.081, *n* = 653) was significant. Looking deeper into the matter of cognitive impairment, logistic regression analysis (Table 3) revealed that—in comparison with no or mild cognitive CI—moderate cognitive impairment significantly increases the likelihood of item nonresponse. The observed OR were 2.78 (CI_95%_ 1.82–4.25, *p* < 0.001) for item nonresponse in general, 3.83 (CI_95%_ 2.58–5.66, *p* < 0.001) for INR more than 5%, and 4.31 (CI_95%_ 2.78–6.68, *p* < 0.001) for INR more than 10%, respectively. Level of cognitive function, age, and gender of NHR, and the duration of the interview were entered in one step as subjective variables in the regression model (Table 3). Sample dummy was introduced for further control of an influence of pre- and post-test sample (i.e. before and after intervention). Referring to this, the non-significant result does not support the assumption that the proportion of INR was influenced by differing pre- and post-test sample characteristics or the main studies’ interventions. Stepwise multiple regression supports above stated INRs’ negative distinctive correlation with MMSE score (β ≈ − 0.40, *p* < 0.001), and positive but weak correlation with age (β ≈ 0.12, *p* < 0.001). The two predictors ‘level of cognitive function’ and ‘age of NHR’ explain a significant proportion of variance in item nonresponse rates (R^2^_corr_ ≈ 16.7%, F ≈ 64.7, *p* < 0.001, *n* = 637).

**Table 3:** Effects of MMSE, age, gender, and interview duration on item nonresponse

Spearman (r_s_) correlation and eta^2^ (ɲ^2^) statistics^a^				
	Duration*INR (r_s_)	Age*INR (r_s_)	Gender*INR (ɲ^2^)	MMSE*INR (r_s_)
r_s_/ɲ^2^, *p* (n)	−0.067, *p* ≈ 0.089 (640)	0.161, *p* < 0.001 (653)	0.004, *p* ≈ 0.081 (653)	−0.355, *p* < 0.001 (655)
BLR – Binary logistic regression statistics^a^ *(factor: MMSE 10–17* vs. *18–30, n = 655)*				
Response	B (SE)	Wald	OR [CI 95%]	p
> 0% INR	1.02	22.4	2.78 [1.82–4.25]	*p* < 0.001
> 5% INR	1.34	45.2	3.83 [2.58–5.66]	*p* < 0.001
> 10% INR	1.46	42.6	4.31 [2.78–6.68]	*p* < 0.001
MLR – Multiple linear regression statistics^a^ *(enter-model, response: INR, n = 637)*				
Factor	B (SE)	β	t	p
Duration	0.00 (0.00)	0.04	1.22	*p* ≈ 0.233
Age	0.14 (0.04)	0.12	3.22	*p* ≈ 0.001
MMSE	−0.65 (0.06)	−0.39	−10.82	*p* < 0.001
Gender	−0.92 (0.78)	− 0.04	−1.17	*p* ≈ 0.242
Sample	−0.77 (0.66)	−0.02	− 0.60	*p* ≈ 0.244
R^2^_corr_	16.5%			
F, p	32.79, *p* < 0.001			
MLR – Multiple linear regression statistics^a^ *(stepwise-model, response: INR, n = 637)*				
Factor	B (SE)	β	t	p
MMSE	−0.65 (0.06)	−0.40	−10.79	*p* < 0.001
Age	0.14 (0.04)	0.12	3.20	*p* < 0.001
R^2^_corr_	16.7%			
F, p	64.71, *p* < 0.001			

*MMSE* Mini-Mental State Examination Score, *INR* Item nonresponse, Duration Interview duration, Sample Pre−/post-test-dummy, *n* Sample sizes, *B* Unstandardized coefficients, *SE* Standard error, *OR* Odds ratio, *β* Standardized coefficient, *t* t-statistic, R^2^_corr_ Adjusted determination coefficient; *F* F-statistic; ^a^ Number of test correction = 14 (α_corr_ = 0.00357, Bonferroni); ^b^ Excluded in step 2: duration, gender, sample

## Discussion

This study examined the quality of a survey regarding pain situation and management in a sample of nursing home residents with no/mild and moderate cognitive impairment. Item nonresponse was used as an indicator for the quality of responses. Our results indicate that the cognitive function is an essential predictor of INR. While age showed significant but very weak, and duration of the interview as well as gender of residents revealed no significant influence on INR, distinct dependencies of INR on cognitive impairment were detected. Residents with decreased cognitive functions exhibit higher rates of INR. Examination of differences in INR between NHR with no or mild and NHR with moderate cognitive impairments revealed statistically medium to large effects (δ = 0.73–0.82): NHR with moderate impairment had explicitly higher INR than NHR with no or mild impairment. The likelihood to feature an INR of 5% (i.e. 10%) or higher for NHR with an MMSE score between 10 to 17 is 3.8-times (i.e. 4.3-times) of that for NHR with an MMSE score between 18 and 30.

Our findings are predominantly in line with the available empirical evidence. Elderly persons with (age-associated) lower cognitive abilities exhibit a poorer response quality, which is consistent with former findings from studies regarding age [7, 29–35] or cognitive function as predictors of survey quality [4, 33, 36–39]. In our study, gender of NHR shows no significant effect on INR, although others reported opposite results [31–33]. Interview duration has sometimes been supposed to affect data quality in terms of obtained response or completion rates [40]. Our data do not support this assumption for face-to-face surveys of NHR. This seems to be to some extent compatible with another study finding [38] that questionnaires’ completion times were not predicted by the cognitive status—cognitive status being supposed as the mainly explanatory factor here at play. The appraisal, which extent of item nonresponse should be seen as a crucial threat to data quality, varies between disciplines and paradigms and has to be interpreted against the specific background of respective field of research, naturally. However, it seems legitimate to critically question information of respondents, i.e. state a risk for data accuracy, if item nonresponse rates of 5% are considerably exceeded [21, 23, 41]. In this present study the descriptive proportions of INR illustrate remarkable losses in information and let us assume that also even moderate cognitive impairment poses a threat to the quality of responses and precision of aggregated results in such samples: One quarter of residents with no or mild cognitive impairment were affected by at least 5% item nonresponse. In comparison, more than half of NHR with moderate cognitive impairment had at least 5% INR. Such high rates are similar to some [31, 38] but not all [36, 42] screened studies conducted in nursing homes. While the impact of item nonresponse on data quality in general has still not yet received exhaustive attention in scientific discussion, explicit research of item nonresponse in surveys of institutionalized people with cognitive impairment is basically almost lacking. Thus, comparison of our findings to others is difficult due to varying study populations, survey modes, or study settings. Additionally, in many studies age or educational status of respondent are used to indirectly measure effects of cognitive impairment.

Our results carefully call the quality of surveys in residents with moderate cognitive impairment into question. Declines in cognitive function [10, 11], i.e. shortcomings in language abilities, short-term and working memory, or restricted concentration and communication abilities are likely to interfere with the question-answer-process (i.e. interpretation, retrieval, judgment, format, and edit) in surveys of NHR. Provision of valid answers to questions premises that respondents successfully search for the most relevant information in mind. If respondents are not able to comply with such a cognitively demanding task and if the search process for relevant information is not executed optimally, the propensity of so called survey satisficing [8] is high. More precisely, this would mean that respondents do not run through the different cognitive stages of the question-answer-process in the most sophisticated way and provide satisfactory instead of optimal answers, which results in response effects or even in incomplete data. Nursing home populations may particularly be prone to such satisficing tendencies and here, item nonresponse serves as a distinct indicator for satisficing in surveys. The consequences of elevated nonresponse rates can be serious and far reaching. INR poses a significant threat for data quality, biasing point estimators, decreasing the statistical precision of interval estimates due to reduced effective sample sizes, and potentially confounding correlation and variance analyses. Systematic biases may be induced when results from survey research in elderly persons aren’t being controlled for the cognitive status or at least be compared between different levels of cognitive impairment. As one consequence, observed differences in distributions or study outcomes may be interpreted as substantial effects while in fact being artificially introduced by simple methodological constraints.

### Limitations and implications for future studies

Some limitations of the study must be acknowledged. While we have every reason to trust that the observed population has been and still is typical of that of nursing homes in general, we cannot rule out the possibility of sampling bias. Unit nonresponse may be higher in men than in women, which affects survey participation propensity. Gender effects may not only be moderated by the grade of cognitive function but cognitive impairment may express itself differently in male and female residents. In a more general perspective, the consequences of cognitive impairment but also the brains’ abilities to cope with damage and structural loss seem to differ inter-individually. Next to illness-related and psychosocial factors, such differences in compensating are frequently attributed to manifestations of cognitive reserve—i.e. resources to maintain cognitive functions on a relatively high level despite neurodegeneration and dysfunctions of the brain [43–45]. One assumption is that individuals with high reserve are able to compensate better for brain damage than those with less reserve. Successful, healthy ageing may protect against late-life mental health problems like depression and is assumed to postpone clinical symptoms as well as to slow down the pathological trajectories of age-associated cognitive decline. Cognitive reserve may also confound and modify individuals’ performance in the question-answer-process despite clinically assessed symptoms or documented forms of progressed dementia. Future studies should control for earlier life course characteristics predicting successful ageing in terms of cognitive resources (e.g. education, occupational attainment, intelligence quotient, life-style indicators). Turning to possible effects of design and survey characteristics on item nonresponse, interview duration showed almost no measurable influence on item nonresponse rates. This may be due to the fact that our interviewers were trained to take their time with interviewing, offering and taking interview breaks when residents’ concentration and alertness were fading. Such—according to our experience—indispensable design proceedings probably enhanced the overall questionnaire completeness rates but may have suppressed or blurred further methodologically signifying response effects. Speaking of interviewers, we cannot rule out that they knowingly or unknowingly deviated from the specified standardized interviewing approach. Future studies about data accuracy, survey quality, and item nonresponse should take possible interviewer effects focusing on interviewer characteristics, behavior, attitudes or knowledge into account. Furthermore, the satisficing mechanism explaining item nonresponse is thought to be a function of cognitive ability, item difficulty, and respondent motivation. Upcoming studies with experimental approaches should not only apply differentiated measures of cognitive ability (e.g. word fluency, reasoning, memory performance) but deliberately include questions of varying difficulty and further formal question conditions. Respondents’ motivation to participate and to provide optimal answers is linked to question content and the subjective topic relevance. Although we can assume this motivational factor to be quite high due to the fact that NHR are reporting their experienced pain situation, we did not measure topic relevance or respondents’ motivation explicitly. Some statistical limitations should be taken into account. Analyses were performed using the combined pre−/posttest data. A moderate share of residents (*n* = 103) was interviewed both pre- and posttest. It was anticipated that intra-individual posttest item nonresponse measures are not related to pretest item nonresponse measures and data were treated as independent samples. To rule out habituation effects or effects due to question, item or topic familiarity, all analyses were repeated either introducing repeated measures indication as control or analyzing all models separately (i.e. split by dependent/independent sample). No differences in substantial results and conclusions were found [see Additional file 4: Sensitivity analyses]. Another possible limitation concerns the nested structure of the data as residents are clustered within nursing homes. To check on clustering effects, fixed effect models eliminating between-cluster variation using nursing home dummy coding were applied. These supplementary sensitivity analyses demonstrated mostly identical results indicating that possible clustering does not affect item nonresponse propensity [see Additional file 4: Sensitivity analyses]. Further important recommendations for future studies include expanded study settings (e.g. acute vs. chronic care or nursing home vs. hospital, outpatient care service), the usage of random sampling, interpenetrated, experimental designs as well as sufficient sample sizes enabling sophisticated multi-level analyses.

## Conclusions

While various phenomena in populations of institutionalized elderly people are gaining more and more attention in geriatric, medical, or nursing research and practice, theory formation and development of specifically adapted methods and instruments lag behind. We would like to conclude that the methodological considerations and our empirical results clearly do support the need for a multidisciplinary ‘general theory’ of the question-answer-process which has to be also inclusive for cognitively impaired elderly persons. Sophisticated research is needed in order to understand underlying information-processing logics of old and oldest old in surveys and to avoid possible ageism tendencies—namely a systematic exclusion of elderly persons with cognitive impairments from health services research and societal decision-making.

## Additional files

Additional file 1: **Table S1.** Comparisons of sample characteristics classified by null-hypothesis families. (PDF 146 kb)

Additional file 2: **Table S2.** Characteristics of item nonresponse distribution. (PDF 120 kb)

Additional file 3: Questionnaire (English version of the applied questionnaire: question text and answer categories). (PDF 196 kb)

Additional file 4: Sensitivity analyses (Additional analyses controlling for possible habituation and cluster effects reveal no substantial differences in study findings). (PDF 376 kb)

## Data Availability

The data are available from the corresponding author upon reasonable request. For the content of the used questionnaire please see ‘Additional file 3: Questionnaire’.

## Abbreviations

ANCOVA: Analysis of Covariance
BLR: Binary logistic regression
CA: Cannot be answered
CAPI: Computer-assisted personal interviewing
CI: Cognitive impairment
CI_95%_: 95% confidence interval
DK: Don’t know
EQ-5D-3 L: Euroqol Quality of Life (5 dimensions, 3 levels)
INR: Item nonresponse
IQR: Interquartile range
MLR: Multiple linear regression
MMSE: Mini-Mental Status Examination
NH: Nursing home
NHR: Nursing home residents
OR: Odds ratio
SD: Standard deviation
VRS: Verbal Rating Scale
