# Supplementary material for: Effects of age and cognitive function on data quality of standardized surveys in nursing home populations
Source: BMC Geriatr. 2019 Sep 3;19:244. doi: 10.1186/s12877-019-1258-0 (PMC6724313; doi:10.1186/s12877-019-1258-0)
Supplement: Supplementary file 1 — Table S1. Comparisons of sample characteristics classified by null-hypothesis families. (PDF 146 kb) [file 12877_2019_1258_MOESM1_ESM.pdf]

Table S1: Comparisons of sample characteristics classified by null-hypothesis families

**Characteristics:**	**Comparison of distributions**			
**Null-hypothesis family I (k=8, α_{corr}=0.00625): MMSE-groups within evaluation**				
	Pre		*Post*	
Female	χ^2≈0.001	p≈0.977 (n=285)	χ^2≈0.346	p≈0.556 (n=372)
Age[a]	t≈-1.213 (df=282)	p≈0.226 (n=284) $CI_{95\%}$ [-3.56, 0.84]	t≈-0.153 (df=371)	p≈0.879 (n=373) $CI_{95\%}$ [-1.89, 1.62]
Duration of Stay[a]	t≈-1.216 (df_{corr}=74)	p≈0.228 (n=271) $CI_{95\%}$ [-2.51, 0.61]	t≈0.919 (df=367)	p≈0.358 (n=369) $CI_{95\%}$ [-0.54, 1.48]
Duration of Interview[b]	t≈0.044 (df=273)	p≈0.965 (n=275) $CI_{95\%}$ [-0:08, 0:08]	t≈-2.856 (df_{corr}=270)	p≈0.005 (n=365) $CI_{95\%}$ [0:03, 0:20]
**Null-hypothesis family II (k=8, α_{corr}=0.00625): Pre- and post-test within MMSE-groups**				
	MMSE 18-30		*MMSE 10-17*	
Female	χ^2≈2.068	p≈0.150 (n=517)	χ^2≈0.115	p≈0.734 (n=140)
Age (years)	t≈-2.674 (df_{corr}=447)	p≈0.008 (n=517) $CI_{95\%}$ [-3.09, -0.47]	t≈-0.451 (df=138)	p≈0.653 (n=140) $CI_{95\%}$ [-3.02, 1.89]
Duration of Stay (years)	t≈0.562 (df=501)	p≈0.574 (n=503) $CI_{95\%}$ [-0.53, 0.96]	t≈2.052 (df_{corr}=78)	p≈0.043 (n=137) $CI_{95\%}$ [0.05, 3.22]
Duration of Interview (min.)	t≈-2.300 (df_{corr}=415)	p≈0.022 (n=506) $CI_{95\%}$ [-0:14, -0:01]	t≈0.630 (df=132)	p≈0.530 (n=134) $CI_{95\%}$ [-0:07, 0:15]
**Null-hypothesis family III (k=4, α_{corr}=0.0125): Pre- and post-test within entire sample**				
Female	χ^2≈2.043	p≈0.153 (n=657)		
Age (years)	t≈-2.586 (df_{corr}=578)	p≈0.010 (n=657) $CI_{95\%}$ [-2.67, -0.37]		
Duration of Stay (years)	t≈1.539 (df=638)	p≈0.124 (n=640) $CI_{95\%}$ [-0.14, 1.18]		
Duration of Interview (minutes)	t≈-1.825 (df_{corr}=602)	p≈0.068 (n=640) $CI_{95\%}$ [-0:11, 0:01]		

Notes: MMSE Mini-Mental State Examination; [a] Years; [b] Minutes; k Number of inferential tests; n Sample sizes; df Degrees of freedom; $CI_{95\%}$ 95% Confidence interval; χ^2 Pearson Chi²; t Student's t

© The Authors
