# Supplementary material for: Effects of age and cognitive function on data quality of standardized surveys in nursing home populations
Source: BMC Geriatr. 2019 Sep 3;19:244. doi: 10.1186/s12877-019-1258-0 (PMC6724313; doi:10.1186/s12877-019-1258-0)
Supplement: Supplementary file 2 — Table S2. Characteristics of item nonresponse distribution. (PDF 120 kb) [file 12877_2019_1258_MOESM2_ESM.pdf]

Table S2: Characteristics of item nonresponse distribution

Descriptive statistics for item nonresponse rate

INR – univariate measures and proportions

	n	Range	Mean (SD)	Median	IQR	Sk, Ku	>0% INR	>5% INR	>10% INR
Pre	286	0-70.4%	6.05% (9.5)	3.84%	0-8.7%	3.5, 17.2[a]	57.7% (f=165)	33.8% (f=97)	18.7% (f=53)
Post	373	0-91.3%	5.40% (8.6)	3.86%	0-8.0%	4.5, 32.8[a]	56.3% (f=210)	31.0% (f=116)	15.6% (f=58)
Total	659	0-91.3%	5.68% (8.9)	3.85%	0-8.3%	3.9, 24.6[a]	56.9% (f=375)	32.2% (f=213)	16.9% (f=111)

Notes: INR Item nonresponse, SD Standard deviation, IQR Interquartile range, Sk Skewness, Ku Kurtosis, f Frequency; [a] Right-skewed, leptokurtic distribution

© The Authors
