# Supplementary material for: Effects of age and cognitive function on data quality of standardized surveys in nursing home populations
Source: BMC Geriatr. 2019 Sep 3;19:244. doi: 10.1186/s12877-019-1258-0 (PMC6724313; doi:10.1186/s12877-019-1258-0)
Supplement: Supplementary file 3 — Questionnaire (English version of the applied questionnaire: question text and answer categories). (PDF 196 kb) [file 12877_2019_1258_MOESM3_ESM.pdf]

Kutschar P., Weichbold M. & Osterbrink J. Effect of Age and Cognitive Impairment on Data Quality of Standardized Surveys in Nursing Home Populations: English Version of the applied Questionnaire

**Question text (Translated from German)**	**Answer Categories / Scale**
**1** In which situations do you experience pain?	Lying or sitting / Getting up / Walking / Bandage change / Urinating / Bowel movement / Washing or personal hygiene / Eating or swallowing / Others | *I don't have pain / don't know / cannot be answered*
**1a** For how long have you been experiencing pain?	Less than or equal to three months / More than three months / More than six months / More than a year | *don't know / cannot be answered*
**1b** In what areas do you feel pain? Please indicate the area that hurts the most.	Body scheme format (pain drawing) | *don't know / cannot be answered*
**2** Please rate the intensity of your pain while you are resting.	I don't have pain / mild pain / moderate pain / severe pain / unbearable pain | *don't know / cannot be answered*
**3** Please rate the intensity of your pain while you are under stress (e.g. moving, coughing).	I don't have pain / mild pain / moderate pain / severe pain / unbearable pain | *don't know / cannot be answered*
**4** During the last three days, were you asked if you were experiencing pain?	Yes / No | *don't know / cannot be answered*
**5** Do you report to the nursing staff when in pain?	Yes, every time / Sometimes / no | *don't know / cannot be answered*
**5a** Did you receive any treatment for your pain, if you did report to the nursing staff?	Yes, every time / Sometimes / no | *don't know / cannot be answered*
**6** Have you received any pain medication in the last three days?	Yes / No | *don't know / cannot be answered*
**6a** How did the pain medication help with your pain?	Very well / Fair / Very poor | *don't know / cannot be answered*
**7** Did you sleep well in the last three nights?	Very well / Fair / Very poor | *don't know / cannot be answered*
**7a** If you did not sleep well, was it due to your pain?	Yes / Some part / No | *don't know / cannot be answered*
**8a** Have you experienced nausea in the last three days?	Yes / No | *don't know / cannot be answered*
**8b** Have you experienced the urge to vomit in the last three days?	Yes / No | *don't know / cannot be answered*
**8c** Have you experienced dizziness in the last three days?	Yes / No | *don't know / cannot be answered*
**8d** Have you experienced breathing difficulties in the last three days?	Yes / No | *don't know / cannot be answered*
**8e** Have you experienced constipation in the last three days?	Yes / No | *don't know / cannot be answered*
**9** Have you received any pain treatment besides pain medication?	Yes / No | *don't know / cannot be answered*
**9a** If yes, did the nursing staff apply cold packs?	Yes / No | *don't know / cannot be answered*
**9b** If yes, did the nursing staff apply heat packs?	Yes / No | *don't know / cannot be answered*
**9c** If yes, did you have a focused conversation about your pain with the nursing staff?	Yes / No | *don't know / cannot be answered*
**9d** If yes, did the nursing staff help you get into a pain relieving position?	Yes / No | *don't know / cannot be answered*
**9e** If yes, did the nursing staff show you pain reducing movements?	Yes / No | *don't know / cannot be answered*
**10** How satisfied are you with the treatment of your pain?	I don't have pain / very satisfied / satisfied / dissatisfied / very dissatisfied | *don't know / cannot be answered*
**10a** Please elaborate.	Open answer format | *don't know / cannot be answered*
**11a-e** EQ-5D-3L Descriptive system (mobility, self-care, usual activities, pain discomfort, anxiety/depression)	The EuroQol Group (1990). EuroQol-a new facility for the measurement of health-related quality of life. Health Policy 16(3):199-208<br>Web page: https://euroqol.org/eq-5d-instruments/eq-5d-3l-about/
**12** EQ Visual analogue scale (self-rated health state)	
**13** Compared to my overall health in the last 12 months, my health in the last three days was…	Better / Same / Worse | *don't know / cannot be answered*

© The Authors
