# Supplementary material for: Effects of age and cognitive function on data quality of standardized surveys in nursing home populations
Source: BMC Geriatr. 2019 Sep 3;19:244. doi: 10.1186/s12877-019-1258-0 (PMC6724313; doi:10.1186/s12877-019-1258-0)
Supplement: Supplementary file 4 — Sensitivity analyses (Additional analyses controlling for possible habituation and cluster effects reveal no substantial differences in study findings). (PDF 376 kb) [file 12877_2019_1258_MOESM4_ESM.pdf]

I. Sensitivity analyses: Question/Survey familiarity ('habituation') effect in NHR who participated both in pre- and posttest

I. a. Adjusted comparisons of item nonresponse rates (ANCOVA)

Sample	Cognitive impairment	Mean (SE, n_{valid})	Mean diff. (SE) [95% CI]	η^2 (F), p
Post	No or mild (MMSE 18-30)	3.93% (0.5, n=289)	7.31% (1.1) [5.22, 9.40]	11.7% (47.5) p<0.001
	Moderate (MMSE 10-17)	11.24% (0.9, n=75)		
$Post^{SENS}$	No or mild (MMSE 18-30)	3.93% (0.5, n=289)	7.30% (1.1) [5.20, 9.39]	11.6% (47.0) p<0.001
	Moderate (MMSE 10-17)	11.23% (0.9, n=75)		
Total	No or mild (MMSE 18-30)	3.98% (0.4, n=502)	7.67% (0.8) [6.10, 9.24]	12.9% (92.4) p<0.001
	Moderate (MMSE 10-17)	11.85% (0.7, n=134)		
$Total^{SENS}$	No or mild (MMSE 18-30)	3.98% (0.4, n=502)	7.68% (0.8) [6.11, 9.24]	12.9% (92.3) p<0.001
	Moderate (MMSE 10-17)	11.65% (0.7, n=134)		

Notes: SENS=further dummy control included (dependent sample: 'no', 'yes')

I. b. Regression models (enter-model, response: INR)

BLR – Binary logistic regression statistics[a] *(factor: MMSE 10-17 vs. 18-30, n=655)*

Response	B (SE)	Wald	OR [CI 95%]	p
>0% INR	1.02	22.4	2.78 [1.82-4.25]	p<0.001
>0% INR^{SENS}	1.03	22.5	2.79 [1.83-4.27]	p<0.001
>5% INR	1.34	45.2	3.83 [2.58-5.66]	p<0.001
>5% INR^{SENS}	1.36	45.6	3.88 [2.62-5.74]	p<0.001
>10% INR	1.46	42.6	4.31 [2.78-6.68]	p<0.001
>10% INR^{SENS}	1.47	42.6	4.31 [2.78-6.68]	p<0.001

MLR – Multiple linear regression statistics[a] *(stepwise-model, response: INR, n=637)*

Factor	B (SE)	β	t	p
Duration	0.00 (0.00)	0.04	1.22	p≈0.233
Duration[SENS]	0.00 (0.00)	0.04	1.18	p≈0.237
Age	0.14 (0.04)	0.12	3.22	p≈0.001
Age[SENS]	0.14 (0.05)	0.11	3.01	p≈0.003
MMSE	-0.65 (0.06)	-0.39	-10.82	p<0.001
MMSE[SENS]	-0.65 (0.06)	-0.39	-10.68	p<0.001
Gender	-0.92 (0.78)	-0.04	-1.17	p≈0.242
Gender[SENS]	-0.88 (0.79)	-0.04	-1.12	p≈0.261
Sample	-0.77 (0.66)	-0.02	-0.60	p≈0.244
Sample[SENS]	-0.81 (0.66)	-0.05	-1.22	p≈0.222
R^2_{corr}	16.5%	R^2_{corr}[SENS]	16.5%	
F, p	32.79, p<0.001	F, p[SENS]	21.92, p<0.001	

Notes: SENS=further dummy control included (dependent sample: ‘no’, ‘yes’)

I. c. t-tests (i.e. mean differences in INR between repeated measure ‘no’ (independent sample) / ‘yes’ (dependent sample))

**Sample**	**Repeated measure (no/yes)**	**Mean (SE, n_{valid})**	**Mean diff. (SE) [95% CI]**	**p**
Total	Independent	5.79% (0.4, n=450)	0.33% (0.8) [-1.16, 1.82]	0.663
	Dependent (repeated)	5.45% (0.6, n=205)		
Posttest	Independent	5.57% (0.5, n=268)	0.64% (0.9) [-1.33, 2.60]	0.525
	Dependent (repeated)	4.94% (0.8, n=103)		
MMSE no/mild, Posttest	Independent	4.17% (0.4, n=210)	0.81% (0.7) [-0.52, 2.13]	0.232
	Dependent (repeated)	3.37% (0.5, n=84)		

MMSE moderate, Posttest	Independent	10.67% (1.9, n=58)	-1.24% (3.9) [-9.02, 6.53]	0.751
	Dependent (repeated)#	11.91% (3.1, n=19)		

M-W-U 493.00; z=-0.692; p=0.489

Notes: Comparisons of mean INR between independent and dependent samples

I. d. Repetition of manuscripts' main analyses split by repeated measures ('no'/'yes'), summary

INR by MMSE-group (ANCOVA)	
Independent	Mean diff. 7.51%*** (4.12% vs. 11.62%)
Dependent	Mean diff. 8.09%*** (3.67% vs. 11.77%)
BLR (factor MMSE 10-17 vs. 18-30)	
Independent	$OR^{>0\%}$=3.26***, $OR^{>5\%}$=4.19***, $OR^{>10\%}$=4.41***
Dependent	$OR^{>0\%}$=2.11**, $OR^{>5\%}$=3.30***, $OR^{>10\%}$=4.10***
MLR (stepwise, response: INR)	
Independent	β^{MMSE}=-0.396***, β^{Age}=0.136**
Dependent	β^{MMSE}=-0.364***, β^{Age}=0.160**

*** p<0.001, ** p<0.01

Notes: summarized findings, not shown in full detail

II. Sensitivity analyses: Cluster sampling – Fixed Effects models eliminating between-cluster variation (NH-dummy adjustment)

II. a. Adjusted comparisons of item nonresponse rates (ANCOVA)

No or mild (MMSE 18-30)	4.03% (0.4, n=502)	7.45% (0.8) [5.85, 9.06]	11.9% (83.33) p<0.001
Moderate (MMSE 10-17)	11.48% (0.7, n=134)		

Notes: Adjustment of between-cluster variance by including n-1 nursing home dummy variables

II. b. Multiple linear regression (MLR)

Factor	B (SE)	β	t	p
Duration	0.00 (0.00)	0.03	0.68	0.495
Age	0.12 (0.05)	0.10	2.48	0.013

MMSE	-0.66 (0.06)	-0.39	-10.45	<0.001
Gender	-0.59 (0.79)	-0.03	-0.75	0.456
Sample	-0.52 (0.66)	-0.03	-0.78	0.436
R^2_{corr}	16.3%			
F, p	19.96, p<0.001			

Notes: Adjustment of between-cluster variance by including n-1 nursing home dummy variables

Abbreviations

NHR	Nursing Home Residents
ANCOVA	Analyses of Covariance
SE	Standard Error
95% CI	95% Confidence Interval
n	Sample Size
F	F-value (ANCOVA)
p	p-value
η^2	Eta-square
MMSE	Mini-Mental Status Examination
Post	Posttest
SENS	Sensitivity Analyses
INR	Item nonresponse rate
BLR	Binary Logistic Regression
B	Unstandardized Coefficient
β	Beta (Standardized Coefficient
OR	Odds Ratio
t	t-value (t-test)
R^2	R-square
corr	Corrected (MLR)
MLR	Multiple Linear Regression
M-W-U	Mann-Whitney U-Test
z	z-value
diff.	Difference (i.e. Mean Difference)
